# Piglet Morphology: Indicators of Neonatal Viability?

**DOI:** 10.3390/ani12050658

**Published:** 2022-03-05

**Authors:** Bryony S. Tucker, Kiro R. Petrovski, Jessica R. Craig, Rebecca S. Morrison, Robert J. Smits, Roy N. Kirkwood

**Affiliations:** 1 School of Animal and Veterinary Sciences, The University of Adelaide, Roseworthy, SA 5371, Australia; kiro.petrovski@adelaide.edu.au (K.R.P.); roy.kirkwood@adelaide.edu.au (R.N.K.); 2 Davies Livestock Research Centre, The University of Adelaide, Roseworthy, SA 5371, Australia; 3 Research and Innovation, Rivalea Australia Pty Ltd., Corowa, NSW 2646, Australia; jcraig@rivalea.com.au (J.R.C.); rmorrison@rivalea.com.au (R.S.M.); 4 Research and Innovation, Australian Pork Limited, Barton, ACT 2600, Australia; rob.smits@australianpork.com.au

**Keywords:** abdominal circumference, crown-to-rump length, low birthweight, piglets, pre-weaning survival, proportion

## Abstract

**Simple Summary:**

Early identification of poor-performing and non-viable piglets is important for effective interventions. Weight has been the consistently used indicator of likely survival in commercial production. We found that piglet survival increased with increasing abdominal circumference (girth) and crown to rump length. When a piglet was proportionately long and wide, they were more likely to be heavier at 24 h and survive until weaning, unless they were a small piglet. Small piglets that were disproportionate were more likely to survive than the proportionate small piglets, especially when they consumed more than 200 g of colostrum. We suggest that the girth and length of the piglet should be used when making production decisions for small piglets.

**Abstract:**

The morphological measures, crown-to-rump length (CR), and abdominal circumference (AC) have been suggested to be as good, if not better, than birth weight for predicting piglet performance. We explored the relationships between CR and AC, and piglet weights at birth and 24 h, to investigate their predictive value for piglet survival. Piglet weight and AC at birth and 24 h, and CR at 24 h were recorded for 373 piglets born to 31 sows. Morphological measures were categorised into two levels for weight and three levels for AC and CR. Further, AC and CR groupings were concatenated to create a new variable (PigProp) to describe the proportionality of piglet morphology. Proportionate piglets had equal CR and AC levels, and disproportionate piglets had contrasting levels. Birth AC was a good predictor of colostrum intake (*p* < 0.001) when accounting for birth weight, but 24 h weight and PigProp were good indicators of actual colostrum intake (*p* < 0.001 for both). The significant interaction of colostrum and PigProp showed that within the smaller piglet groups, those who had greater than 200 g of colostrum had higher 24 h weight and survival (*p* < 0.001 both). As expected, as body weight and colostrum intake increased, so did weight change to d 21 (P = 0.03 and trend at *p* = 0.1, respectively). A similar pattern was seen with increasing PigProp group (*p* < 0.001); however, piglets from the disproportionate group 1,3 had the greatest observed weight change (5.15 ± 0.06 kg). Our data show morphological measures may be more predictive of piglet viability in terms of both performance and survival than weight and there may be subgroups that have higher than expected chances of survival.

## 1. Introduction

Pre-weaning piglet mortality is a prevalent economic and welfare issue for the swine industry [1,2]. Despite extensive research on ways to improve the survival of piglets using genetic selection, environmental improvements, and management routines, pre-weaning mortality remains high [3,4,5]. Multiple factors interact to influence a piglet’s ability to survive, including their birth weight, colostrum intake, and viability at birth, as well as factors inherent to the sow, such as her gestation environment and her post-partum management [2,6,7]. 

Selection for larger litter sizes in recent years has increased the within litter birth weight variation and, consequently, competition between littermates for colostrum in the first 24 h of life [7,8,9,10,11]. Furthermore, often a sow’s teat number is inadequate to support such a large litter without interventions such as supplemental colostrum and (or) milk for her piglets. For these reasons, although selection for larger litter sizes has resulted in a higher number of piglets weaned per sow, it has also contributed to a greater incidence of pre-weaning mortality [12].

Piglet birth weight is a production indicator for pre-weaning growth and survival with lower birth weight piglets underperforming [9,13,14,15]. However, recent research has raised questions as to the utility of using only birth weight [7], which in commercial settings is usually estimated visually, as a criterion for informing management decisions such as fostering [16,17]. Other more specific measures of piglet morphology at birth could serve as potential early life indicators for pre-weaning performance and survival.

Recent studies have suggested that a piglet’s morphology may be more indicative of performance [16,17,18,19] than birth weight alone. A key paper by Douglas et al. [19] found that piglet crown to rump (CR) length, abdominal circumference (AC), and body mass index are good predictors of growth performance, although they rely heavily on body weight and the age of the piglet. However, that study did not investigate if these same morphological measures could be used to indicate survival. Since that study, to our knowledge, no other paper has considered these morphological measures as indicators of performance. This is surprising as these morphological measures are used commonly in humans to estimate foetal development and childhood growth potential [20,21]. Interestingly, among other factors, human ethnicity can influence gestation day foetal and neonatal measures, such as estimated weight, long bone length, and AC, with racial/ethnic-specific measures providing greater accuracy [22]. The authors surmised that, like in humans, these morphological measures may be different across pig genetic lines and countries. The previous study was conducted in the UK where the average litter size was 13.6 piglets per litter [19,23]. This is greater than the reported Australian litter sizes of between 11.1 and 12.9 piglets per litter [24]. It is well known that with increased litter size uterine space becomes limiting and piglet variation increase [9,10]. Therefore, it is not unreasonable to assume that the morphological measures as indicators of performance and survival would be different for a herd with lower litter size. Further, the present study focuses on how morphology influences performance of different weight piglets, as opposed to across the population, in conjunction with weight in addition to its prediction of survival. 

Therefore, the present study tested the suggestion that measures of neonatal piglet morphology such as AC and CR are indicative of a piglets’ chance of survival to weaning under Australian commercial conditions. It was hypothesised that a longer crown to rump length indicates likely improved survival while a shorter abdominal circumference indicates a lower potential colostrum consumption and, thus, compromised survival.

## 2. Materials and Methods

### 2.1. Experimental Design

All experimental sows farrowed over a 3 d interval, having 452 live and 38 stillborn piglets in total. From the piglets born alive, 79 piglets were not considered for inclusion within the trial due to the time of birth being outside of observation hours. At farrowing, a total of 373 piglets born to 31 mixed parity sows (birth parities 2 to 9) were selected for inclusion in the study from litters that were born between 0700 h and 2000 h, for the collection of morphological measures.

Sow parity, day of farrowing, and estimated piglet birth time by visual observation were recorded. Within 5 min of birth, piglets were sexed, ear-tagged, weighed, and had their abdominal circumference (AC) recorded. The AC was determined using a standard measuring tape to an accuracy of 0.5 cm on the basis of the smallest clear measure on the measuring tape at the widest point around the piglet’s trunk, posterior to the umbilicus [19]. Once AC had been measured, the piglets were placed back with their sow under a heat lamp in the creep area, and no additional assistance other than standard production protocols was provided. 

At 23 to 26 h after delivery of the first piglet, all piglets were individually weighed, and their crown-rump length (CR) and AC were recorded. The CR was measured from the base of the tail to the crown of the head using the same measuring tape as for AC. The morphological measures were performed by the same technical staff for the duration of the experiment to reduce individual bias. All mortalities and removals were recorded from birth until weaning on day 21 (± 2 d). Piglets were individually weighed at weaning.

### 2.2. Animals and Housing

Sows were housed in groups of 40 sows per pen from mating until confirmation of pregnancy at day 28 of gestation via ultrasound, following which sows were moved to pens of 80 sows per pen equipped with electronic feeders supplying 2.5 kg/d of a standard gestation feed formulated to provide 13.8 MJ digestible energy (DE)/kg, 14.3% crude protein (CP), and 0.4% standardised ileal digestible (SID) lysine. Sows were moved into individual slatted floor farrowing crates at 110 d (± 2 d) gestation and a fed standard lactation diet, formulated to provide 15 MJ DE/kg, 16.7% protein and 0.90% SID lysine, at 3.8 kg from entry to farrowing and ad libitum from farrowing until weaning, with any spoiled feed removed daily. Sows were monitored daily for general health and welfare throughout the study. Each farrowing crate was equipped with sow and piglet level nipple drinkers, a solid floored creep area from the back to the middle side of the crate with a heat lamp positioned centrally over the creep. All sows farrowed without induction. No sow on trial required manual assistance with farrowing as per production protocol (farrowing assistance provided if sows showed signs of distress during farrowing and or if 45 min had elapsed from birth of the last piglet with no farrowing progress evident). 

Minimal fostering occurred, with stock people moving only non-tagged pigs, when necessary (within the first day), on to sows not on trial. Piglets were processed at day 4, receiving an iron injection, tail docking, and toltrazuril (Baycox®; Bayer Animal Health, Manheim, Germany) drench.

### 2.3. Statistical Analysis

All statistical analyses were performed using SAS version 9.4 (Statistical Analysis Software, Cary, NC, USA). The variables were categorised as follows: AC (3 levels) and CR (3 levels) at birth, and 24 h where applicable (Table 1). Weight was originally categorised into groupings of 5 levels; however, this was later modified, and the data were pooled into 2 groups on the basis of the analysis showing no differences between weight classes > 1.1 kg. The 24 h AC and CR groups were concatenated (CR level, AC level) to create a new variable grouping, Piglet Proportions (PigProp: 9 levels: Table 1), where proportionate piglets had equal CR and AC levels and disproportionate piglets had contrasting levels. Colostrum intake (CI) was calculated using the equation developed by Devillers, et al. [25]:

$$CI=-217.4+0.217\times t+1861019\times \frac{W}{t}+BW\times \left(\frac{54.8-1861,019}{t}\right)\times (0.9985-3.7\times 10^{-7}\times t_{fs}^{2})$$

where *CI* = colostrum intake (g), *W* = piglet body weight at 24 h (kg), *BW* = piglet body weight at birth (kg), *t* = age (min), and *t_fs_* = time elapsed from birth to first sucking (min). On the basis of Devillers, et al. [25] research, *t_fs_* is assumed to be 30 min and t is 1440 min.

Colostrum intake was also categorised into 2 levels (CIc) on the basis of 200 g being the recommended minimum amount of colostrum needed to survive (level 1 includes piglets who consumed <200 g of colostrum and level 2 includes piglets who consumed ≥200 g).

Correlations between measures at birth and 24 h were tested using PROC CORR with the output being the Pearson’s correlation coefficient and the respective 95% confidence intervals. Correlation was considered to be very high if *r* ≥ 0.90, high if *r* = 0.7 to 0.89, moderate if *r* = 0.5 to 0.69, low if *r* = 0.3 to 0.49, and negligible if *r* < 0.3 [26]. 

The effect of PigProp category on 24 h weights was estimated using a mixed model in PROC MIXED, as presented in Equation (1):

(1) $$24\;weight=CIc_{af,bo,ls,p,s}\times PigProp_{af,bo,lg,ls,p,s}$$

where *fr* = farrowing room, *bo* = birth order, *gl* = gestation length, *ls* = litter size, *p* = parity, and *s* = sow.

The effect of birth weight category and PigProp category on weight change was estimated using a Mixed model in PROC MIXED, as presented in Equation (2):

(2) $$Weight\;change=BWc_{af,bo,gl,p,s}+PigProp_{afbo,gl,p,s}+CIc_{afbo,gl,p,s}$$

where *fr* = farrowing room, *bo* = birth order, *gl* = gestation length, *ls* = litter size, *p* = parity, and *s* = sow.

The effect of birth weight and birth AC on colostrum intake was estimated using a mixed model in PROC MIXED, as follows:

Birth factors predicting colostrum intake, Equation (3):

(3) $$Colostrum\;intake\;\left(g\right)=BWc_{af,bo,gl,p,s}+BACc_{af,bo,gl,p,s}$$

Twenty-four hour factors indicating colostrum intake, Equation (4):

(4) $$Colostrum\;intake\;\left(g\right)=24Wc_{af,bo,gl,p,s}+PigProp_{af,bo,gl,p,s}$$

where *fr* = farrowing room, *bo* = birth order, *gl* = gestation length, *ls* = litter size, *p* = parity, and *s* = sow.

For all mixed models, piglet sex was tested in the preliminary model but removed as nonsignificant. The outputs were the means and their respective standard errors. Significance was assessed at the *p* = 0.05 level.

The effect of PigProp on survival from day 1 to day 21 (weaning) was estimated using a linear regression in PROC GLIMMIX as presented in Equation (5):

(5) $$Survival={\left(CIc\times PigProp\right)}_{gl,s}$$

where *gl* = gestation length and *s* = sow. Farrowing room, parity, litter size, birth order, and sex were tested in the preliminary model but were found to be not significant and as such removed from the final model. The outputs of all linear regressions were geometric means and their respective 95% confidence intervals.

## 3. Results

The analysed dataset included 373 piglets born alive within observation hours. The mean stillborn rate was 1.22 piglets per litter and the mean total litter size was 14.6 (±2.89) piglets per litter. Twenty-five experimental piglets died between birth and 24 h, and an additional 56 piglets died between 24 h and weaning. Therefore, the analysis included the records of 373 piglets born alive of which 348 survived to 24 h and 292 to weaning (d 21). Mean colostrum intake was estimated at 218.2 ± 167.5 g, and the mean litter size at 24 h was 11.6 ± 3.2 piglets. Descriptives of morphological measures within the dataset displayed in Table 2.

The distribution of piglets across the concatenated CR and AC groupings at 24 h showed that more piglets were born with proportionate CR and AC sizes than disproportionate (Table 1). The least likely to occur were highly disproportionate piglets with high CR and low AC (group 3,1) or low CR and high AC (group 1,3). 

### 3.1. Correlation of Morphological Measures

All individual birth and 24 h measures were positively correlated, with a moderate to a very high degree of correlation occurring between measures (Table 3). A very high degree of correlation between birth and 24 h weight and moderate correlation between 24 h CR and AC at birth, as well as at 24 h, were observed. AC change from birth to 24 h showed mostly negligible correlations with piglet measures, except a moderate correlation with 24 h AC.

### 3.2. 24 h Weight

**Figure 1:**
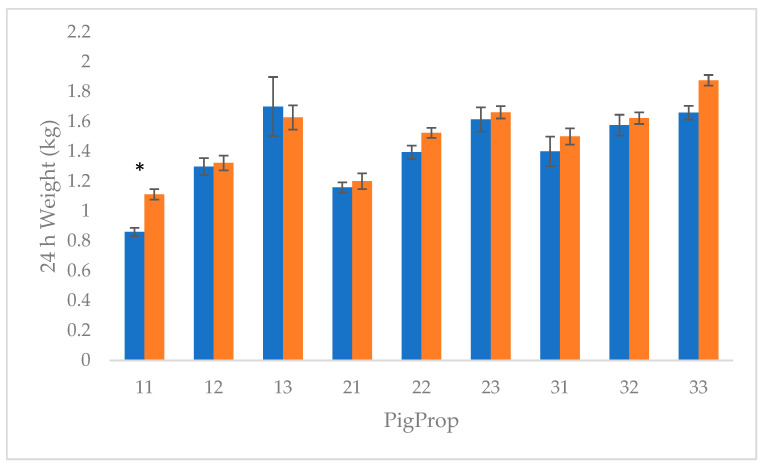
Mean ± standard error for 24 h weight for 348 piglet records for colostrum intake category PigProp interaction. Blue represents piglets who consumed < 200 g colostrum. Orange represents piglets who consumed ≥ 200 g colostrum. Adjusted for farrowing room, birth order, gestation length, litter size, sow, and parity. * denotes a significant difference at *p* < 0.05 within the PigProp group.

All piglets showed increasing mean 24 h weights when they obtained greater than 200 g of colostrum, regardless of PigProp grouping, except 1,3 (*p* < 0.001; Figure 1). This difference is greatest in 1,1 piglets having significantly higher 24 h weight when they consumed more colostrum (*p* < 0.001).

### 3.3. t Change, 24 h to Weaning

Piglet weight change increased as birth weight and colostrum intake increased (*p* = 0.03 and 0.13, respectively; Table 4). In pigs that were from PigProp 1,1, 1,2, or 2,1, weight change was significantly lower (*p* < 0.001). Piglets from 1,3 showed the highest weight change. Piglets in group 2,2 and higher showed a weight change greater than 4.30 kg. 

### 3.4. Colostrum Intake

As birth weight and AC increased, so did the predicted potential colostrum intake (*p* < 0.001 for both; Table 5). Colostrum intake was higher in pigs with greater 24 h weight (*p* < 0.001; Table 6). As PigProp increased, so did colostrum intake, whereas, within the shortest pigs, those with larger AC consumed more colostrum (higher degree of disproportion) (Table 5 and Table 6). 

### 3.5. Survival

Survival from 24 h to d 21 was significantly affected by the interaction between PigProp and colostrum intake (*p* < 0.001). When piglets consumed more than 200 g of colostrum, their survival increased substantially (Table 7). No piglets from groups 1,3 and 2,3 who consumed <200 g of colostrum died in this data set. Most improvement was observed for 1,1 piglets who consumed >200 g (*p* < 0.001). The mean survival also showed a similar increase for piglets in 1,2, 2,1, and 3,1 groupings when more colostrum was consumed. 

## 4. Discussion

We hypothesised that a longer CR length was associated with improved survival while a shorter AC was associated with lower potential colostrum/milk consumption, and thus compromised survival. On the basis of our data, we can partially accept our hypothesis. As overall piglet size increased (piglet proportions), so did colostrum intake and survival. Proportionate pigs showed increased survival as size increased and had less variation in survival than disproportionate pigs. 

The degree of proportion or disproportion could be a better indicator for survival and performance than morphological measures alone.

### 4.1. Abdominal Circumference 

Piglet AC had a linear relationship with body weight at both birth and 24 h, as previously documented for humans [27,28]. The high correlation observed between AC and weight may be related to successful suckling and post-natal consumption of colostrum and milk. Human infant AC was negatively correlated with time from the last feeding, which supports the linear relationship but also highlights the possible variability and low correlation with AC across time [29]. Although not measured in the present study, a similar relationship between time from feeding and AC in piglets may occur. Colostrum intake showed a negligible correlation with birth AC but an improved low correlation with 24 h AC. This correlation may improve if true time from last suckle was adjusted for. The design of this study only included those piglets born under observation, thus allowing for accurate recording at 24 h of age. Further, as the number of milk letdowns and successful suckling bouts were not recorded, the utility of these results is limited. If a strong relationship is present when true time from last suckle and or number of suckles is accounted for, AC change from birth to 24 h after birth could be used as a strong indicator for the successful consumption of sufficient colostrum [30]. 

Within this experiment, we assumed a 30 min interval from last suckle for each piglet and found that AC at birth when combined with birth weight was a good indicator of potential colostrum intake. These findings agree with the work of Douglas et al. [19], who showed that AC and birth weight were good predictors of performance. This is likely due to them being indicative of stomach capacity, which is greater in larger pigs allowing more milk and colostrum to be consumed at each let down [31,32]. Further, our second colostrum model supports this assumption as 24 h weight was a good measure of consumed colostrum when modelled with PigProp. As expected, when 24 h weight increased, so did colostrum intake. However, AC only influenced colostrum intake through PigProp and not individually. In light of these results, the true individual time from last suckle may have greater effect on smaller piglets.

In practice, the reproducibility of birth AC may be higher for prediction of colostrum intake than birth weight as it is unlikely to be as affected by factors difficult to account for like umbilicus (and its fluid) weight, although both measures cannot avoid influence by retained birth fluid. This could potentially distort the weight and or presumed empty stomach size; however, we assume this effect would be similar for both measures.

Although birth AC was a good indicator of potential colostrum intake, it could be significantly impacted by successful suckling and, as such, would require greater care for timing when measuring and applying in practice. AC at birth or 24 h alone did not influence survival, although its impact on colostrum intake, which does influence survival, suggests it should not be discounted as an indirect measure of survival as well as potential performance. 

### 4.2. Crown to Rump Length

CR increased linearly with AC and weight at 24 h with a moderate correlation observed. It is commonplace in humans to use CR length during pregnancy and at birth in conjunction with AC, head circumference, and weight to gauge foetal development [20,21]. Interestingly, CR alone did not significantly influence weight, colostrum intake, or survival, contrary to our hypothesis. It was assumed that in larger litters with reduced individual uterine space, there would be greater variation in piglet size, and as a result, more piglets with shorter CR would be present and a difference would be observed. The lack of influence of CR preweaning is supported by the work of Douglas et al. [19], who found CR had no significant predictive value prior to weaning. However, our relatively small sample size may have precluded detection of a CR effect, which would explain the lack of litter impact. Unfortunately, CR was not measured at birth to reduce handling time immediately following piglet expulsion; however, it would have been interesting to compare the survival of piglets with different AC and CR at birth to those at 24 h, the former being a period of relatively higher piglet mortality [3,4]. 

It can be assumed that CR is not influenced by time from the last suckle, supported by its negligible correlation to colostrum intake, making it a more reproducible measure than AC and affected by less circumstantial factors. However, even in humans, CR is rarely used alone, often being combined with a waist to height ratio to diagnose foetal, infant, and childhood conditions such as growth retardation and obesity [33,34,35]. The previous pig morphology paper showed that CR can be used to calculate BMI, which was also a good performance indicator pre- and post-weaning. Therefore, CR is a good indicator of growth but could be a better survival indicator if combined with other morphological measures or used to calculate them.

### 4.3. Piglet Proportions (PigProp)

Previous studies have demonstrated that piglet body shape rather than birth weight had a greater influence on piglet performance [16,17,19], which was further expanded in this experiment for survival. The AC alone could be significantly affected by colostrum and (or) milk intake, while CR is not influenced by colostrum intake, which is critical to survival outcomes. 

When considered together (i.e., PigProp), an interesting pattern of distribution across the groupings and survival was apparent. Proportionate piglets, being those that had the same grouped CR and AC (1,1; 2,2; 3,3 as defined in Table 1), occurred more frequently (53%) than the disproportionate groupings, with the greatest number being the smallest piglets. It is important to note that disproportion did not occur due to feeding and colostrum intake but was an important factor in determining survival. It could be suggested that the level of disproportion is an indicator of uterine growth performance such as intrauterine growth restriction (IUGR), which is well known to impact piglet viability at birth [18]. This is supported by the knowledge that as IUGR severity increases, CR decreases; however, it is not known if this effect remains when it is considered relative to AC. 

Within the population, 12% of piglets were extremely disproportionate (1,3 and 3,1), resulting in a high error in the analysis. Therefore, we are unable to confidently determine what impact being highly disproportionate has on survival in comparison to more proportionate pigs. However, the remaining disproportionate piglets showed that having a slightly larger AC or CR can improve survival significantly in smaller pigs. As hypothesised, the smallest proportional piglets survived significantly less than did the other proportionate piglets and the disproportionate piglets. Although the influence of PigProp on colostrum intake was known, the survival difference within the smallest piglet grouping when more than 200 g of colostrum was consumed was greater than expected. Although subjective, we observed visually that differentiating between these slightly disproportionate piglets at 24 h was very difficult, lending support to the suggestion that accurate measuring of piglets at birth is valuable in determining viability.

As AC category increased within the same CR grouping, colostrum intake increased. A similar pattern was observed for increasing CR length but was not as definitive. Surprisingly, the piglets from 1,3 had the highest colostrum intake of all PigProp groupings, similar to the largest piglets. Bootstrapping was applied to test if this would remain true in a theoretical larger population, and this was supported. The authors suggest that this may have been due to these piglets having a disproportionately larger stomach capacity, and being able to obtain a full stomach early on provided them greater energy to outcompete their similar-sized poor-performing littermates. However, as stated above, the number of suckling bouts or a controlled allocation/administration of fluids was not tested in this study, and therefore relation to stomach capacity cannot be confirmed. Regardless, PigProp at 24 h may be used as an indirect measure of colostrum intake and continual feeding of the piglet.

The smallest proportionate piglets showed similar weight change to the disproportionate piglets of groups 1,2 and 2,1, and piglets from groups 1,3 showed similar weights to piglets from groupings 2,2 and greater. From these data, we can infer that within visually smaller piglet groupings usually designated as poor performers and survivors, there are piglets that have survival and growth rates similar to those of larger piglets. It would be interesting to further investigate a larger population on the basis of these findings to determine if within the smallest grouping (1,1), there are characteristics which can define the poorest performers once again without having to estimate or monitor colostrum intake. Surprisingly none of the models or the morphological measures showed influence of sex on survival, which is the same as in humans but contrary to previous research in pigs [36,37]. Sex has been shown to influence a piglet’s chance of survival, with males tending to die from being overlain by the sow more than do females [37]. This sex effect was not evident in our study, although the suggested sex effect may be influenced more by the sex ratio of the litter rather than sex per se, as suggested by Seyfang et al. [38]. A larger investigation into morphological measures involving sex within litter effects would provide further evidence of the applicability of AC and CR as a tool for predicting survival and growth.

Although there is a standard growth chart for humans that is based on recurring measures or multiple measure types, there is not one for pigs, and it is known that there is a large variation in height and weight in humans due to race [39]. Although speculative, a similar degree of variation may exist between pig breeds for these morphological measures [21]. It is beyond the scope of this study to compare this; however, in comparison to studies from other countries, our mean CR and AC were smaller. Our mean CR and AC were similar to the means reported in light-weight pigs (AC = 23.3 ± 1.52 cm and CR = 24.0 ± 1.91) in the study by Douglas et al. [19]. However, we are unable to determine if this was due to uterine capacity as litter size was not reported. Despite this, another study did report greater mean CR (28 ± 0.26 cm) than our study, despite having higher mean litter size (16 ± 1.17) [40]. It is reasonable to assume that longer piglets need more space and thus with greater litter size, the capacity to reach this would be reduced. The disparity between our and other studies’ findings further strengthen the argument that the variation seen between human races is replicated in pigs. Our findings support the hypothesis that not all small pigs will die, as even within the smallest poor-performing piglet group, there are still piglets that if they obtained sufficient colostrum can survive to weaning without additional intervention.

## 5. Conclusions

Current management decisions are based on the abundance of literature supporting the strong relationship between birth weight and pre-weaning growth and survival. Low birth weight piglets are treated as a uniform group assumed to underperform without additional intervention. The present study suggests that there may be more indicators of the potential risk to small piglets than just their weight, and that there may be subgroups that have higher than expected chances of survival. Disproportionately small piglets (either CR or AC one category higher than the other) show a much higher likelihood of survival than proportionally small piglets. However, these findings may be somewhat limited by the sample size as the effect for piglets greatly disproportionate (AC and CR in opposing extreme categories) could not be determined as they were born much less frequently. This strengthens the argument posed by previous research that birthweight alone may not be sufficient for an indication of performance and survival and novel measures such as AC and CR may be useful to assist with management decisions for post-natal piglet management when used in concert. 

## Figures and Tables

**Table 1:** Definition of categorised variables of interest: piglet body weight (W) and abdominal circumference (AC) at birth, and piglet body weight, crown to rump length (CR), and Piglet proportions (PigProp) at 24 h.

Morphological Measures		Birth		24 h	
	Group	N	Range	N	Range
W (kg)	1	103	0.50–1.10	86	0.50–1.10
	2	270	1.12–2.20	262	1.12–2.40
AC (cm)	1	131	16.0–22.0	145	18.0–25.0
	2	141	22.5–25.0	117	25.0–27.0
	3	101	25.5–30.0	86	27.0–32.0
CR (cm)	1	-	-	116	17.0–23.5
	2	-	-	131	24.0–26.5
	3	-	-	101	27.0–32.0
	**Group**	**N**	**CR group (cm)**		**AC group (cm)**
PigProp	1,1	81	17.0–23.5		18.0–25.0
	1,2	28	17.0–23.5		25.0–27.0
	1,3	7	17.0–23.5		27.0–32.0
	2,1	47	24.0–26.5		18.0–25.0
	2,2	55	24.0–26.5		25.0–27.0
	2,3	29	24.0–26.5		27.0–32.0
	3,1	17	27.0–32.0		18.0–25.0
	3,2	34	27.0–32.0		25.0–27.0
	3,3	50	27.0–32.0		27.0–32.0

**Table 2:** Descriptive summary statistics (mean ± SD) of the raw dataset for piglet bodyweight, abdominal circumference (AC), and crown to rump length (CR).

Morphological Measures	Birth	24 h	d 21
N	373	348	292
W (kg)	1.33 ± 0.34	1.38 ± 0.36	6.25 ± 1.61
AC (cm)	23.59 ± 2.63	25.45 ± 2.81	-
CR (cm)	-	24.83 ± 2.98	41.34 ± 4.32

**Table 3:** Pearson’s correlation coefficients and respective 95% confidence intervals for the relationship between morphological measures: bodyweight (BW = birth weight, W = 24 h weight), abdominal circumference (AC) at birth and 24 h, and crown to rump length (CR) at 24 h.

Measures	BW	BAC	W	24 h AC	24 h CR	CI
BAC	72.0 (66.7–76.6)	-				
W	93.6 (92.1–94.8.)	72.1 (66.6–76.8)	-			
24 h AC	77.8 (73.2–81.6)	64.6 (58.1–70.4)	81.9 (78.1–85.1)	-		
24 h CR	72.6 (67.3–77.3)	64.4 (57.8–70.1)	73.1 (67.8–77.7)	65.1 (58.6–70.8)	-	
CI	20.2 (9.9–30.0)	28.9 (18.1 –37.5)	53.4 (45.4–60.5)	41.0 (31.9–49.4)	29.0 (19.0–38.3)	-
AC change	14.5 (4.0–24.6)	−32.8 (−41.9–−23.1)	19.2 (8.90–29.1)	48.7 (40.3–56.3)	7.60 (−2.9–18.0)	18.4 (8.0–28.4)

**Table 4:** Proportion and respective 95% confidence intervals of weight change from 24 h to d 21 for 348 piglets for birth weight category (BW), piglet proportions (PigProp), and colostrum intake. N = number of piglets per group. Adjusted for farrowing room, birth order, gestation length, litter size, parity, and sow.

Measure	Groups		N	Weight Change
BW (kg)	≤1.11		103	4.33 ± 0.31
	>1.12		270	4.88 ± 0.22
Colostrum intake (g)	<200		153	4.47 ± 0.27
	≥200		195	4.74 ± 0.24
PigProp	1,1	Proportionate	81	3.97 ± 0.27 ^A^
	1,2	Disproportionate	28	4.14 ± 0.34 ^AB^
	1,3	Disproportionate	7	5.15 ± 0.56 ^BC^
	2,1	Disproportionate	47	3.93 ± 0.29 ^A^
	2,2	Proportionate	55	4.79 ± 0.28 ^C^
	2,3	Disproportionate	29	5.03 ± 0.34 ^C^
	3,1	Disproportionate	17	4.97 ± 0.40 ^C^
	3,2	Disproportionate	34	4.53 ± 0.34 ^BC^
	3,3	Proportionate	50	4.91 ± 0.31 ^C^

^A–C^ Different superscripts indicate significant differences (*p* < 0.05) between groups.

**Table 5:** Mean ± standard error for predicted colostrum intake in 348 piglets. BW = birth weight category. BAC = birth abdominal circumference category. N = number of piglets per group. Adjusted for farrowing room, sow, litter size, parity, and birth order.

Measure	Groups	N	Colostrum Intake (g)
BW (kg)	≤1.11	86	195.7 ± 28.7
	>1.12	262	222.5 ± 24.7
BAC (cm)	16.0–22.0	119	158.5 ± 26.6 ^A^
	22.5–25.0	132	211.2 ± 27.0 ^B^
	25.5–30.0	97	257.7 ± 29.5 ^C^

^A–C^ Different superscripts indicate significant differences (*p* < 0.05) between groups.

**Table 6:** Mean ± standard error for consumed colostrum intake in 348 piglets. W = 24 h weight. Piglet proportions (PigProp) = concatenated crown to rump length and abdominal circumference categories at 24 h. N = number of piglets per group. Adjusted for farrowing room, birth order, litter size, sow, and parity.

Measure	Groups		N	Colostrum Intake (g)
W (kg)	≤1.11		86	148.3 ± 31.0 ^A^
	>1.12		262	256.0 ± 23.0 ^B^
PigProp	1,1	Proportionate	81	155.1 ± 26.7 ^AB^
	1,2	Disproportionate	28	161.0 ± 34.5 ^ABD^
	1,3	Disproportionate	7	281.4 ± 55.5 ^CE^
	2,1	Disproportionate	47	104.6 ± 28.6 ^A^
	2,2	Proportionate	55	190.5 ± 29.8 ^BC^
	2,3	Disproportionate	29	211.0 ± 34.9 ^BCE^
	3,1	Disproportionate	177	245.5 ± 40.1 ^CE^
	3,2	Disproportionate	34	225.9 ± 33.9 ^CE^
	3,3	Proportionate	50	244.5 ± 31.6 ^E^

^A–E^ Different superscripts indicate significant differences (*p* < 0.05) between groups.

**Table 7:** Proportion and respective 95% confidence intervals of survival rates from 24 h to d 21 for 348 piglets. Piglet proportions (PigProp) = concatenated crown to rump length and abdominal circumference categories at 24 h. N = number of piglets per group. Adjusted for farrowing room, birth order, gestation length, sow, and parity.

PigProp		CI (g)	N	% Survival
1,1	Proportionate	<200	50	48.0 (34.6–61.7)
		≥200	31	90.3 (73.9–96.9)
1,2	Disproportionate	<200	12	83.3 (52.1–95.8)
		≥200	16	87.5 (61.3–96.9)
1,3	Disproportionate	<200	1	100 (95.0–100.0)
		≥200	6	83.3 (36.7–97.7)
2,1	Disproportionate	<200	33	75.8 (58.4–87.4)
		≥200	14	100 (95.0–100)
2,2	Proportionate	<200	20	95.0 (71.6–99.3)
		≥200	35	94.3 (79.8–98.6)
2,3	Disproportionate	<200	6	100 (95.0–100)
		≥200	23	95.7 (74.6–99.4)
3,1	Disproportionate	<200	4	75.0 (23.6–96.7)
		≥200	13	84.6 (54.8–96.1)
3,2	Disproportionate	<200	8	87.5 (98.3–46.1)
		≥200	26	88.5 (69.6–96.2)
3,3	Proportionate	<200	19	94.7 (70.5–99.3)
		≥200	31	93.5 (77.5–98.4)

## Data Availability

The data presented in this study are available on reasonable request from the corresponding author.
